# Osteogenesis-Related Long Noncoding RNA GAS5 as a Novel Biomarker for Osteonecrosis of Femoral Head

**DOI:** 10.3389/fcell.2022.857612

**Published:** 2022-03-22

**Authors:** Guanzhi Liu, Sen Luo, Yutian Lei, Ming Jiao, Ruomu Cao, Huanshuai Guan, Run Tian, Kunzheng Wang, Pei Yang

**Affiliations:** Bone and Joint Surgery Center, Second Affiliated Hospital of Xi’an Jiaotong University, Xi’an, China

**Keywords:** osteonecrosis of femoral head, long noncoding RNA, bioinformatics, biomarkers, osteogenesis

## Abstract

**Background:** The lack of effective biomarkers makes it difficult to achieve early diagnosis and intervention for osteonecrosis of the femoral head (ONFH). Hence, we aimed to identify novel long noncoding RNA (lncRNA) biomarkers for ONFH.

**Methods:** High-throughput RNA sequencing was performed to detect lncRNA and mRNA expression levels in subchondral bone samples from three patients with ONFH and three patients with femoral neck fractures. Integrated bioinformatics analyses were conducted to identify lncRNAs associated with ONFH development and their potential functions and signaling pathways. A co-expression network was constructed based on the gene time-series expression data in GSE113253. After selecting lncRNA GAS5 as a novel biomarker for ONFH, bone marrow mesenchymal stem cell (BMSC) osteogenic differentiation assays were performed to verify the association between lncRNA GAS5 and osteogenic differentiation. Alkaline phosphatase (ALP) staining and quantitative reverse transcription polymerase chain reaction (RT-qPCR) were used to measure the osteogenic phenotype and lncRNA GAS5 expression. Finally, for further validation, ONFH rat models were established, and lncRNA GAS5 expression in subchondral bone was detected by RT-qPCR.

**Results:** We identified 126 and 959 differentially expressed lncRNAs and genes, respectively. lncRNA GAS5 expression level was significantly downregulated in patients with ONFH compared to the control group patients. The BMSC osteogenic differentiation assays showed that ALP activity increased gradually from days 3 to 7, while the lncRNA GAS5 expression level was significantly upregulated in the osteogenic differentiation induction groups. Furthermore, *in vivo* experiments suggested that the bone volume/tissue volume value and trabecular thickness significantly decreased in the ONFH rat model group compared to the control group, whereas the trabecular space significantly increased in the ONFH group compared to the control group. In addition, the lncRNA GAS5 expression level significantly decreased in the ONFH rat model group.

**Conclusion:** The lncRNA GAS5 expression level was highly associated with BMSC osteogenic differentiation and was significantly downregulated in both the subchondral trabecular bone tissue of ONFH patients and ONFH rat models. Therefore, lncRNA GAS5 can serve as an ONFH osteogenic biomarker to provide an effective target for early diagnosis and molecular therapy of ONFH.

## Introduction

Osteonecrosis of the femoral head (ONFH) is a severe and disabling orthopedic disease characterized by aseptic and avascular necrosis of the bone tissue, microfractures of the subchondral bone, and femoral head collapse (Mont et al., 2020; Cohen-Rosenblum and Cui, 2019; Saidi and Magne, 2011) (Mont et al., 2020; Cohen-Rosenblum and Cui, 2019; Saidi and Magne, 2011). It is mainly caused by hip trauma and some non-traumatic etiological factors such as long-term and high-dose glucocorticoid application, alcohol abuse, and coagulopathy (Xian et al., 2020; Ikeuchi et al., 2015). Previous studies suggested that the development of ONFH is associated with microcirculation impairment, decreased bone marrow mesenchymal stem cell (BMSC) osteogenic differentiation, and increased adipogenesis differentiation (Liu et al., 2020; Li et al., 2018; Qi and Zeng, 2015)–(Qi and Zeng, 2015; Li et al., 2018; Liu et al., 2020). However, the precise mechanism of the pathogenesis of ONFH remains to be elucidated. In addition, it is difficult to screen for ONFH in the early phase using routine imaging approaches (Kwang et al., 2008; Arbab and König, 2016). Most patients with hip pain and dysfunction require total hip arthroplasties when it rapidly progresses to femoral head collapse or hip osteoarthritis (Sultan et al., 2019; Boontanapibul et al., 2020). Therefore, the development of novel biomarkers is urgently needed to improve early diagnosis of ONFH and to investigate the molecular mechanisms of ONFH.

Long noncoding RNAs (lncRNAs) are noncoding RNAs (ncRNAs) greater than 200 nucleotides in length that have been suggested to play important roles in various biological processes and diseases (Yang et al., 2020; Wang et al., 2019a; Ouyang et al., 2020) (Yang et al., 2020; Wang et al., 2019a; Ouyang et al., 2020). Some previous studies focused on the lncRNAs that regulate BMSC or osteoblast phenotypes, such as lncRNA RP11‐154D6, lncRNA CRNDE, and lncRNA MALAT1 (Yang et al., 2019; Xiang et al., 2019; Shi et al., 2021)–(Xiang et al., 2019; Yang et al., 2019; Shi et al., 2021). However, more research on lncRNAs associated with ONFH or BMSC phenotypic changes during the onset of ONFH is still needed (Chen et al., 2019; Fu et al., 2021). Hence, the identification and validation of lncRNA biomarkers for ONFH *in vitro* and *in vivo* are necessary to achieve early diagnosis and investigate their molecular mechanisms.

In the present study, we annotated and classified lncRNAs and mRNAs based on the high-throughput RNA sequencing raw data included in our previous studies (Jiao et al., 2021). Their expression levels in the subchondral bone tissue of patients with ONFH and femoral neck fracture were obtained. Using integrated bioinformatics methods, a novel lncRNA biomarker, lncRNA growth arrest-specific transcript 5 (GAS5), was identified. Osteogenic differentiation assays indicated that lncRNA GAS5 expression in BMSCs was significantly upregulated during osteogenic differentiation. Meanwhile, the expression of lncRNA GAS5 was significantly downregulated in the ONFH rat models. Thus, lncRNA GAS5 was highly associated with the development of ONFH and may serve as a diagnostic biomarker for ONFH.

## Materials and Methods

### Clinical Subchondral Bone Sample Collection

The present study was approved by the Ethics Committee of the Second Affiliated Hospital of Xi’an Jiaotong University (Ethical Approval number No. 2019035). All tissue donors provided written informed consent for this study. Finally, subchondral bone samples from six patients who underwent primary total hip arthroplasty (three ONFH patients and three patients in the control group with femoral neck fracture) were obtained from the Second Affiliated Hospital of Xi’an Jiaotong University. All the samples were snap-frozen and stored in liquid nitrogen until further analysis.

### High-Throughput RNA Sequencing

Frozen subchondral bone samples were rapidly ground in liquid nitrogen. Total RNA was extracted by TRIzol reagent (Invitrogen) following the manufacturer’s instructions. The NEBNext Poly(A) mRNA Magnetic Isolation Module (New England Biolabs), RiboZero Magnetic Gold Kit (Illumina), and KAPA Stranded RNA-Seq Library Prep Kit (Illumina) were used to achieve RNA enrichment and sequencing library generation. An Agilent Bioanalyzer 2100 system was used to conduct the quality control analysis. Finally, high-throughput RNA sequencing was performed based on the Illumina HiSeq 6000 sequencing platform (Illumina) using the TruSeq SR Cluster Kit (Illumina).

### lncRNA Identification and Differential Expression Analysis

After trimmed and reads filtering (Solexa pipeline program, Cutadapt software, and FastQC software), further lncRNA and mRNA alignment were both conducted by Hisat2 according to human reference genome indexing (hg38). Then we performed principal component analysis to calculate the heterogeneity between samples and remove the outliers. The differentially expressed lncRNAs (DELs) and differentially expressed mRNAs (DEGs) were defined as lncRNAs or mRNAs with |log2FoldChange| ≥ 1 and a *p* value < 0.05 [calculated by “edgeR” package (Robinson et al., 2009)].

### Enrichment Analysis

Gene Ontology (GO) function enrichment analysis, Kyoto Encyclopedia of Genes and Genomes (KEGG) signaling pathway enrichment analysis, and gene set enrichment analysis were conducted by the “clusterProfiler” package with the threshold set at *p* value < 0.05.

### BMSC Culturing and Osteogenic Differentiation

Human BMSCs were purchased from Procell (CP-H166, China) and cultured in minimum essential medium-α (α-MEM, Gibco, United States) supplemented with 10% fetal bovine serum (FBS, Gibco, United States) at 37°C in a humidified environment containing 5% CO_2_. When the BMSCs reached 70% confluence in six-well plates, osteogenic induction was performed using osteogenic medium (100 nM dexamethasone, 50 μg/ml ascorbic acid, and 10 mM β-glycerophosphate, Sigma-Aldrich).

### Alkaline Phosphatase Staining

We performed alkaline phosphatase (ALP) staining (Yan et al., 2020) on days 3 and 7 after the induction of osteogenic differentiation to measure the osteogenic differentiation phenotype of BMSCs using a BCIP/NBT ALP staining kit (C3206, Beyotime, China). Briefly, the plates were washed with phosphate-buffered saline, fixed with 4% paraformaldehyde, washed with ddH_2_O, ALP stained with BCIP/NBT working solution at room temperature, and washed again with ddH_2_O. Finally, ALP staining image analysis was conducted using ImageJ software.

### Quantitative Real-Time PCR

In this study, we performed reverse transcription using StarScript II First-strand cDNA Synthesis Mix (A223-02, Genestar, China). Subsequently, 2× Universal SYBR Green Fast qPCR mix (RK21203, Abclonal, China) was used to conduct the quantitative real-time PCR (RT-qPCR) according to the manufacturer’s instructions. The results were analyzed by using the 2−ΔΔCt method, and GAPDH was used as an endogenous reference gene to normalize the gene expression data. The sequences of primers are as follows:

Human GAPDH (forward primer GAC​AGT​CAG​CCG​CAT​CTT​CT, reverse primer GCG​CCC​AAT​ACG​ACC​AAA​TC), human GAS5 (forward primer GTT​GTG​TCC​CCA​AGG​AAG​GAT​GAG, reverse primer TGT​CTA​ATG​CCT​GTG​TGC​CAA​TGG), Human RUNX2 (forward primer AGC​AGC​ACT​CCA​TAT​CTC​TAC​TAT, reverse primer CAT​CAG​CGT​CAA​CAC​CAT​CAT), rat GAS5 (forward primer GCA​AGC​TCC​ACA​CAA​GGT​CCT​TC, reverse primer TGT​TCA​AGC​ATC​CAT​CCA​GTC​ACC), rat GAPDH primer (purchased from Sangon Biotech, China, B661204).

### Construction of ONFH Rat Model

All animal experiments included in this study were approved by the Laboratory Animal Care Committee of Xi’an Jiaotong University. A total of 24 adult SPF Sprague–Dawley rats weighing 350–400 g were purchased from the Medical School of Xi’an Jiaotong University Animal Center. The rats were divided into two groups: the ONFH group and the control group. The rats were anesthetized with sodium pentobarbital (50 mg/kg, intraperitoneal injection), the fur was removed, and the skin was sterilized around the surgical area. An incision was made in the hip joint (right side) and then separated by layer to expose the femoral head. The hip joint was dislocated, the round ligament was removed, blood supply to the femoral head was stopped, the wound was disinfected, and then the incision layer was replaced. All the rats were killed after 9 weeks. Six femoral heads on the right side were collected for further micro-CT analysis, and 18 subchondral bone samples from the femoral heads were collected for RNA extraction and quantitative real-time reverse transcription polymerase chain reaction (RT-qPCR).

### Micro-CT

The main bone tissue parameters of the femoral heads were evaluated using high-resolution micro-CT scanning and reconstruction. Finally, the values of bone volume/tissue volume (BV/TV, %) were calculated to reflect the bone mass condition in the femoral heads, and the trabecular thickness (Tb.Th, mm) and trabecular space (Tb.Sp, mm) were calculated to reflect the trabecular structure.

### Statistical Analyses

Independent sample t-tests and Mann–Whitney U tests were conducted for the difference analysis according to the distribution of independent variables. IBM SPSS Statistics 22 software was used to perform all the statistical analyses, and *p* < 0.05 was considered statistically significant.

## Results

### Identification of Differentially Expressed lncRNAs

**FIGURE 1:**
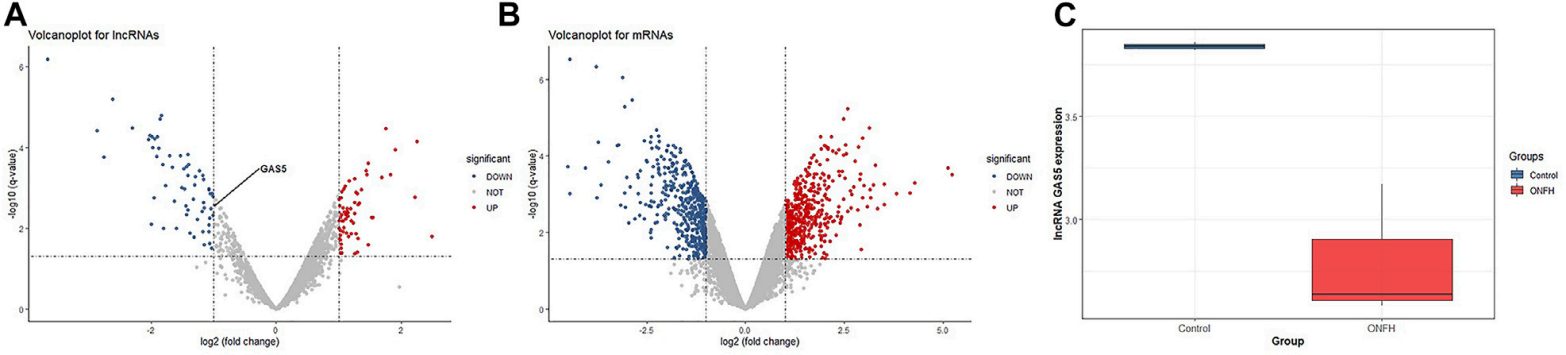
Identification of differentially expressed lncRNAs and mRNAs. **(A)** Volcano plot for lncRNAs. **(B)** Volcano plot for mRNAs. **(C)** lncRNA GAS5 expression level in the ONFH group and control group.

We performed lncRNA and mRNA annotation based on RNA-seq data, and the expression levels of 2966 lncRNAs and 13925 mRNAs were detected in clinical subchondral bone samples in this study. One sample was recognized as an outlier sample and removed through principal component analysis (PCA, shown in Supplementary Files S1, S2). Then, according to the thresholds as |log2FoldChange| ≥ 1 and *p* value < 0.05, a total of 126 differentially expressed lncRNAs (DELs) and 959 differentially expressed mRNA (DEGs) were identified (Figures 1A,B). Additionally, our results in Figure 1C showed that the lncRNA GAS5 expression level of ONFH patients was significantly different from that of the control group patients (log2FoldChange < −1 and *p* value = 0.003).

### Function and Signaling Pathway Enrichment Analyses

**FIGURE 2:**
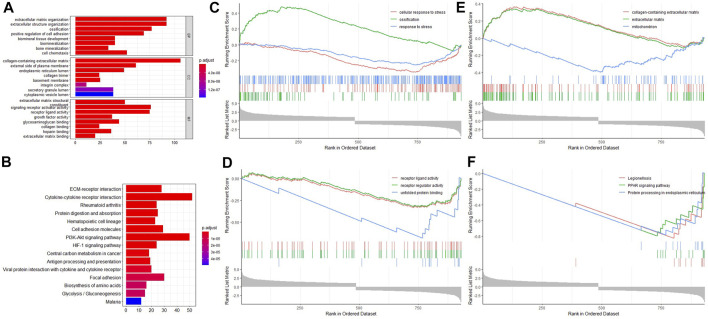
Enrichment analyses for differentially expressed lncRNAs and differentially expressed mRNAs. **(A)** GO enrichment analysis. **(B)** KEGG signaling pathway enrichment analysis. **(C)** GSEA biology process enrichment analysis. **(D)** GSEA molecular function enrichment analysis. **(E)** GSEA cell component enrichment analysis. **(F)** GSEA KEGG signaling pathway enrichment analysis.

GO function enrichment analysis indicated that these DELs and DEGs were highly associated with biological processes like extracellular matrix organization, ossification, and bone mineralization; molecular functions like extracellular matrix structural constituent and signaling receptor activator activity; and cell components like the collagen-containing extracellular matrix and the external side of the plasma membrane. In addition, KEGG signaling pathway enrichment analysis suggested that these DELs and DEGs may play roles in signaling pathways like the PI3K-Akt signaling pathway, the HIF-1 signaling pathway, and cytokine–cytokine receptor interaction (Figures 2A,B). In order to improve the accuracy of enrichment analysis, we further performed GSEA analysis for all these DELs and DEGs. The results showed that they were involved in ossification processes, receptor regulator activity, and PPAR signaling pathways (Figures 2C–F). Therefore, it is necessary to investigate the association between lncRNA GAS5 expression and BMSC osteogenic differentiation.

### lncRNA GAS5 Co-Expression Analysis and Interaction Prediction Analysis

lncRNA and mRNA expression profiles during BMSC osteogenic differentiation periods (0 h, 4 h, 1 day, 3 days, 7 days, 14 days) in GSE113253 datasets were downloaded from the GEO database (Rauch et al., 2019). We further calculated the co-expression correlation between the novel biomarker lncRNA GAS5 and mRNAs. In total, 683 co-expression genes were identified based on the thresholds of |Pearson correlation coefficient| > 0.9 and *p* < 0.05 (Supplementary File S3). Then, an osteogenesis-associated gene list consisting of 399 genes (including ossification, osteoblast differentiation, and osteoblast proliferation) was obtained from the MsigDB database (Supplementary File S4). The results of intersection analysis in Table 1 showed that 11 osteogenesis-associated genes were co-expressed with lncRNA GAS5 (PTPN11, HNRNPC, CCDC47, DHX9, HSPE1, FBL, RDH14, TWIST2, PHB, RSL1D1, DHX36). In addition, we performed lncRNA–mRNA interaction analysis and lncRNA–protein interaction analysis based on the RNAInter database (http://www.rnainter.org/) and the AnnoLnc2 database (http://annolnc.gao-lab.org/) to investigate the potential functional route of GAS5. In total, 1870 lncRNA GAS5–mRNA interaction pairs and 523 lncRNA GAS5–protein interaction pairs were obtained (Supplementary Files S5, S6).

**TABLE 1:** lncRNA GAS5 and osteogenesis genes co-expression data (*n*=11).

lncRNA	Osteogenesis gene	Pearson correlations	*p* value
GAS5	PTPN11	0.92	<0.001
GAS5	HNRNPC	0.90	<0.001
GAS5	CCDC47	0.92	<0.001
GAS5	DHX9	0.90	<0.001
GAS5	HSPE1	0.91	<0.001
GAS5	FBL	0.92	<0.001
GAS5	RDH14	0.93	<0.001
GAS5	TWIST2	0.92	<0.001
GAS5	PHB	0.91	<0.001
GAS5	RSL1D1	0.95	<0.001
GAS5	DHX36	0.94	<0.001

### The Association Between lncRNA GAS5 and BMSC Osteogenic Differentiation

**FIGURE 3:**
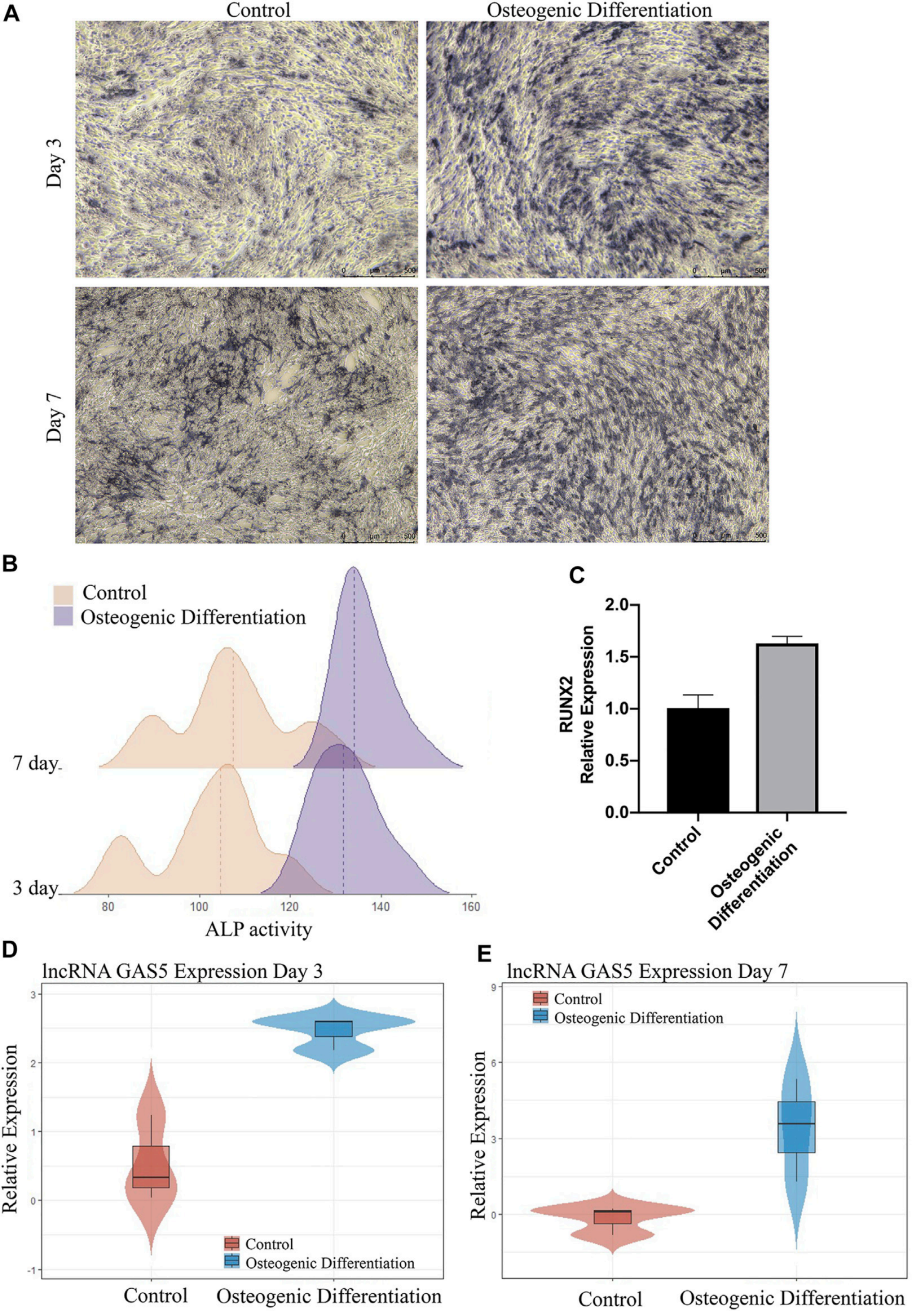
Association between lncRNA GAS5 and BMSC osteogenic differentiation. **(A)** ALP staining on days 3 and 7 of osteogenic differentiation induction group and control group. **(B)** Ridge plot for ALP activity. **(C)** RUNX2 expression levels on day 7 of the osteogenic differentiation induction group and control group. **(D,E)** lncRNA GAS5 expression level on days 3 and 7 after BMSC osteogenic differentiation induction (or control culture).

The expression data of lncRNA GAS5 in GSE113253 revealed its expression trend during the osteogenic differentiation of BMSCs. However, analysis of the differences in lncRNA GAS5 expression levels between osteogenic differentiation induction groups and normal proliferation medium groups can make these results more convincing. Therefore, in this study, we conducted an ALP staining assay and RT-qPCR on days 3 and 7 after the induction of BMSC osteogenic differentiation to verify the association between lncRNA GAS5 and the development of the BMSC osteogenic phenotype. ALP activity increased gradually from day 3 to day 7, with the ALP activity in the osteogenic differentiation induction groups being significantly higher than that in the normal proliferation medium groups (Figures 3A,B). The RT-qPCR results in Figure 3C indicated that the expression of osteogenic marker RUNX2 is significantly upregulated in osteogenic differentiation group on day 7 (*p* = 0.002). Finally, lncRNA GAS5 expression levels in the osteogenic differentiation induction and normal control groups were tracked at these two separate osteogenic differentiation points. The results showed that the expression level of lncRNA GAS5 was significantly upregulated (*p* < 0.05) in the osteogenic differentiation induction groups compared to the normal proliferation medium groups (Figures 3D, E).

### lncRNA GAS5 Expression Validation in ONFH Rat Models

**FIGURE 4:**
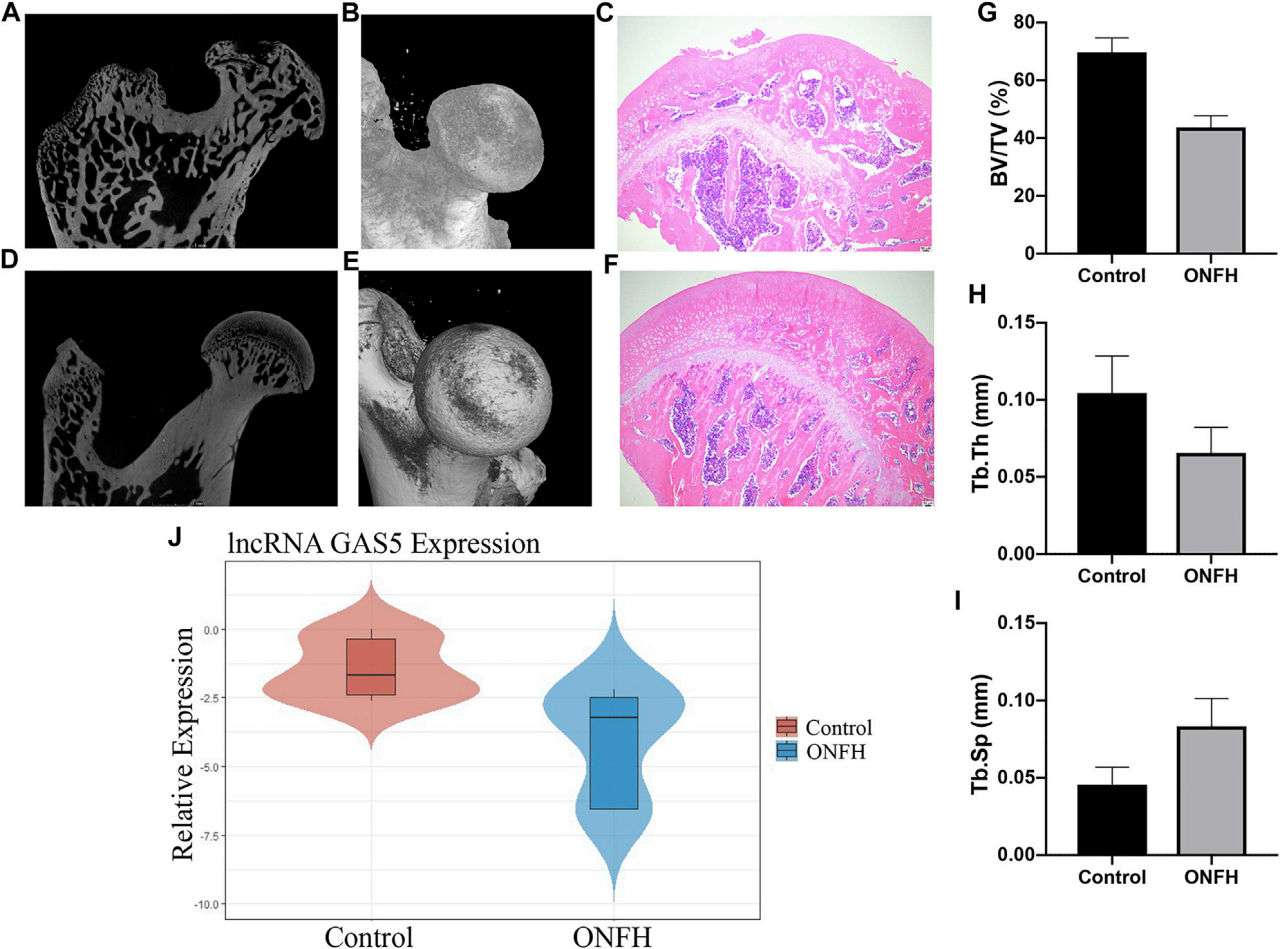
lncRNA GAS5 expression level in ONFH rat model. **(A–F)** Micro-CT and hematoxylin–eosin staining images of femoral head of rat models in the ONFH group and healthy control group. **(G–I)** The BV/TV (%), Tb.Th (mm) values, and Tb.Sp (mm) values of rat femoral heads. **(J)** lncRNA GAS5 expression level in ONFH rat models compared to healthy control rats.

ONFH rat models were constructed, and the main bone tissue parameters of the femoral heads were measured using micro-CT 9 weeks after induction. The subchondral trabecular bone in the weight-bearing area of the femoral head was thinner and sparser according to the results of micro-CT and hematoxylin–eosin staining (Figures 4A–F). In addition, the BV/TV (%) and Tb.Th (mm) values were significantly lower in the ONFH group than in the control group. The value of Tb.Sp (mm) significantly increased in the ONFH group (Figures 4G–I). These parameters showed the changes in the bone mass level and trabecular structure, indicating the development of ONFH. RT-qPCR was performed to evaluate the expression of lncRNA GAS5 in the femoral head subchondral bone tissue. The results showed that lncRNA GAS5 expression was significantly decreased in the ONFH group compared to the control group (Figure 4J), consistent with our findings in clinical femoral head subchondral bone tissue. Therefore, lncRNA GAS5 can act as an osteogenic biomarker for ONFH development.

## Discussion

ONFH is a severe orthopedic disease with disabling symptoms that often necessitates hip replacement (Li et al., 2019; Wu et al., 2019). It can seriously affect the quality of life of patients, particularly young people (Cui and Saleh, 2008; Pascart et al., 2017). However, early diagnosis and targeted therapy for ONFH are still limited because of unclear molecular mechanisms and lack of biomarkers (Fu et al., 2019; Han et al., 2019). Hence, in this study, we performed RNA sequencing (RNA-Seq) analysis and identified that the lncRNA GAS5 is associated with the development of ONFH. Further *in vitro* and *in vivo* experiments were performed to validate whether lncRNA GAS5 plays a role in the BMSC osteogenesis process and whether the expression of lncRNA GAS5 was significantly downregulated with ONFH development in the ONFH rat models. To the best of our knowledge, this is the first study to indicate that the expression level of lncRNA GAS5 changes with ONFH development and that it can act as an ONFH osteogenic biomarker.

It is widely accepted that lncRNAs play important roles in various biological processes and diseases (Ju et al., 2019; Zhang et al., 2018). However, little attention has been focused on the identification of ONFH-associated lncRNA biomarkers, which limited the achievement of ONFH early diagnosis and a deeper understanding of its molecular mechanism (Zhangyang et al., 2018). Therefore, in the current study, through RNA-Seq and bioinformatics analysis, we identified 126 differentially expressed lncRNAs (DELs) and 959 differentially expressed genes (DEGs) in the femoral head subchondral bone samples of the ONFH group compared to the control group. Interestingly, we found that the expression of lncRNA GAS5 was significantly downregulated in patients with ONFH (log_2_FoldChange < −1 and *p* = 0.003). lncRNA GAS5 is associated with angiogenesis and osteogenesis. It has been found that lncRNA GAS5 can regulate angiogenesis and activate the HIF1A/VEGF signaling pathway by binding to TAF15 (Peng et al., 2021). Moreover, Centofanti et al. (2020) suggested that the expression of lncRNA GAS5 is significantly downregulated in osteoblastic cells from patients with osteoporosis (OP) . Moreover, several previous studies have demonstrated that lncRNA GAS5 can play important roles in regulating angiogenesis and BMSC osteogenic differentiation during OP development (Wu et al., 2021; Feng et al., 2019). Wang et al. (2019b) measured GAS5 expression during the osteogenic differentiation process of BMSCs and found that the expression of lncRNA GAS5 was significantly increased during osteogenic differentiation compared to day 0 of osteogenic induction. In the current study, GO function enrichment analyses and KEGG signaling pathway enrichment analyses as well as GSEA analyses indicated these DELs were associated with functions like extracellular matrix organization, ossification, bone mineralization, extracellular matrix structural constituents and pathways like PI3K-Akt signaling pathway, and HIF-1 signaling pathway, and cytokine–cytokine receptor interaction. These function and signaling pathways have suggested that they are highly associated with the development of ONFH and BMSC osteogenesis process by some previous studies (Jing et al., 2020; Jiang et al., 2019; Tingart et al., 2008)–(Tingart et al., 2008; Jiang et al., 2019; Jing et al., 2020). HIF 1α is found to be upregulated in the ischemic side of the femoral head, and it can act as a positive regulator of Sox9 activity in femoral head osteonecrosis (Zhang et al., 2011). In addition, the PI3K/AKT signaling pathway may play a significant role in the pathogenesis of ONFH by regulating the proliferation and apoptosis of osteoblasts and osteoclasts (Deng et al., 2019; Wang et al., 2021). However, analysis of differences in lncRNA GAS5 expression between the osteogenic induction and non-osteogenic induction groups at the same time point would make this more reasonable. Therefore, in this study, we detected lncRNA GAS5 expression in the osteogenic induction group compared to the non-osteogenic induction group at days 3 and 7 after induction (or control intervention). The results showed that lncRNA GAS5 expression levels were significantly upregulated in osteogenic differentiation induction groups compared to the normal proliferation medium groups. In addition, it is not well known whether lncRNA GAS5 expression is associated with ONFH. To verify the BMSC osteogenic differentiation phenotype and lncRNA GAS5 expression changes in ONFH, it is necessary to perform corresponding *in vivo* experiments. Therefore, we established ONFH rat models, measured the osteonecrosis development and osteogenesis changes by micro-CT, and then detected the lncRNA GAS5 expression level by RT-qPCR. Our results suggest that the subchondral trabecular bone in the weight-bearing area of the femoral head became thinner and even collapsed. In addition, micro-CT data showed that BV/TV (%) and Tb.Th (mm) significantly decreased in the ONFH group compared to the control group, whereas Tb.Sp (mm) significantly increased in the ONFH group. Additionally, the lncRNA GAS5 expression level was significantly lower in the ONFH group than in the control group. To the best of our knowledge, this study is the first to identify lncRNA GAS5 as a novel biomarker for ONFH using high-throughput RNA-Seq and integrated bioinformatics analyses while comprehensively validating its expression both *in vitro* and *in vivo*. These results indicate that lncRNA GAS5 can serve as an ONFH osteogenic biomarker, providing an effective target for early diagnosis and molecular therapy of ONFH.

## Conclusion

In conclusion, we identified lncRNA GAS5 as a novel biomarker for ONFH which may serve as a potential treatment target using high-throughput RNA-seq and integrated bioinformatics analyses. Comprehensive *in vitro* and *in vivo* validation have been conducted. lncRNA GAS5 expression levels were highly associated with BMSC osteogenic differentiation and were significantly downregulated in ONFH subchondral trabecular bone tissue.

## Data Availability

The datasets presented in this study can be found in online repositories. The names of the repository/repositories and accession number(s) can be found below: ArrayExpress (accession: E-MTAB-11493).
