# Supplementary figures and images for: Osteogenesis-Related Long Noncoding RNA GAS5 as a Novel Biomarker for Osteonecrosis of Femoral Head
Source: Front Cell Dev Biol. 2022 Mar 22;10:857612. doi: 10.3389/fcell.2022.857612 (PMC8980611; doi:10.3389/fcell.2022.857612)

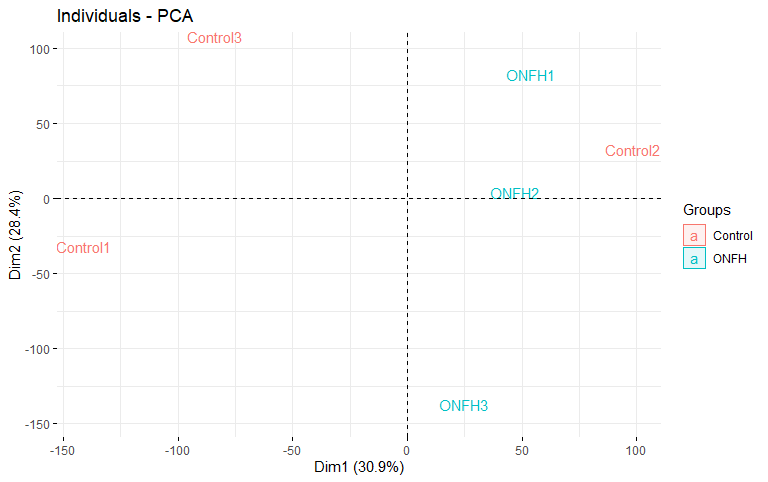

Supplement: Supplementary file 1 [file Image1.TIFF]

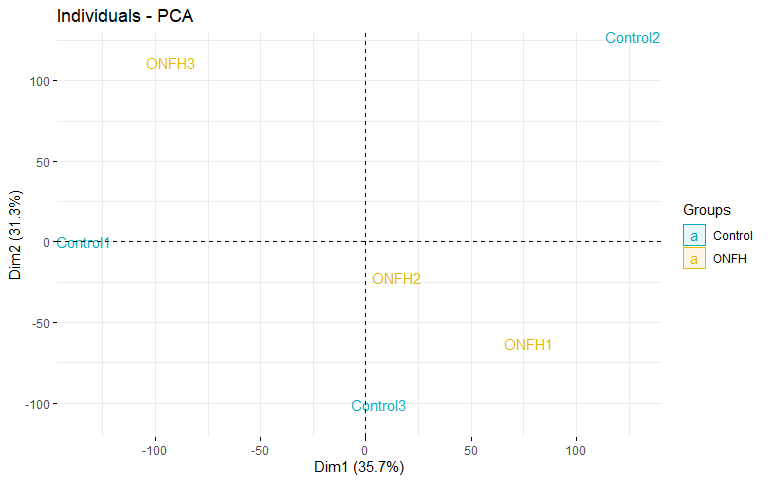

Supplement: Supplementary file 4 [file Image2.TIFF]
